# Delayed Contralateral Nephrectomy Halted Post-Ischemic Renal Fibrosis Progression and Inhibited the Ischemia-Induced Fibromir Upregulation in Mice

**DOI:** 10.3390/biomedicines9070815

**Published:** 2021-07-14

**Authors:** Beáta Róka, Pál Tod, Tamás Kaucsár, Éva Nóra Bukosza, Imre Vörös, Zoltán V. Varga, Balázs Petrovich, Bence Ágg, Péter Ferdinandy, Gábor Szénási, Péter Hamar

**Affiliations:** 1 Institute of Translational Medicine, Semmelweis University, 1094 Budapest, Hungary; beata.roka@gmail.com (B.R.); todpal90@gmail.com (P.T.); kaucsar.tamas@med.semmelweis-univ.hu (T.K.); nora.bukosza@gmail.com (É.N.B.); szenasi.gabor@med.semmelweis-univ.hu (G.S.); 2 Institute for Translational Medicine, Medical School, University of Pécs, 7624 Pécs, Hungary; 3 Department of Pharmacology and Pharmacotherapy, Semmelweis University, 1089 Budapest, Hungary; voros.imre@med.semmelweis-univ.hu (I.V.); varga.zoltan@med.semmelweis-univ.hu (Z.V.V.); balazs.petrovich@gmail.com (B.P.); agg.bence@med.semmelweis-univ.hu (B.Á.); peter.ferdinandy@pharmahungary.com (P.F.); 4 HCEMM-SU Cardiometabolic Immunology Research Group, Semmelweis University, 1089 Budapest, Hungary; 5 Pharmahungary Group, 6722 Szeged, Hungary

**Keywords:** ischemia-reperfusion injury, kidney fibrosis, microRNAs, mice

## Abstract

(1) Background: Ischemia reperfusion (IR) is the leading cause of acute kidney injury (AKI) and results in predisposition to chronic kidney disease. We demonstrated that delayed contralateral nephrectomy (Nx) greatly improved the function of the IR-injured kidney and decelerated fibrosis progression. Our aim was to identify microRNAs (miRNA/miR) involved in this process. (2) Methods: NMRI mice were subjected to 30 min of renal IR and one week later to Nx/sham surgery. The experiments were conducted for 7–28 days after IR. On day 8, multiplex renal miRNA profiling was performed. Expression of nine miRNAs was determined with qPCR at all time points. Based on the target prediction, plexin-A2 and Cd2AP were measured by Western blot. (3) Results: On day 8 after IR, the expression of 20/1195 miRNAs doubled, and 9/13 selected miRNAs were upregulated at all time points. Nx reduced the expression of several ischemia-induced pro-fibrotic miRNAs (fibromirs), such as miR-142a-duplex, miR-146a-5p, miR-199a-duplex, miR-214-3p and miR-223-3p, in the injured kidneys at various time points. Plexin-A2 was upregulated by IR on day 10, while Cd2AP was unchanged. (4) Conclusion: Nx delayed fibrosis progression and decreased the expression of ischemia-induced fibromirs. The protein expression of plexin-A2 and Cd2AP is mainly regulated by factors other than miRNAs.

## 1. Introduction

Renal ischemia reperfusion (IR) is one of the leading causes of acute kidney injury (AKI) [1]. After severe renal tubular injury, maladaptive repair processes are activated that drive renal fibrosis and the development of chronic kidney disease (CKD) [2,3].

Currently, no therapy exists to inhibit the progression of renal fibrosis. Combined inhibition of the renin-angiotensin-aldosterone system is beneficial but does not prevent renal fibrosis and progression to end-stage renal failure [4,5,6]. Serious unilateral renal IR combined with delayed contralateral nephrectomy (Nx) is a relatively new and a promising murine model for studying the protective mechanisms of IR-induced progressive renal fibrosis [7]. Without Nx, the function of the injured kidney was lost within a few weeks [8,9]. Surprisingly, Nx partially restored the function of the IR-injured kidneys and delayed fibrosis progression for several months, making this model suitable for studying the mechanisms of renal recovery following IR injury [7,9]. We demonstrated recently that Nx considerably delayed the progression of fibrosis, as the development of end-stage renal failure was postponed from 14 to 140 days [9]. As this is a relatively new model, the underlying mechanisms of the Nx-induced functional improvement are largely unexplored. The role of reduced hypoxia [10], inflammation and macrophage infiltration [9,10] has been implicated in the beneficial effects of Nx.

Regarding post-transcriptional regulation, the involvement of histone modifications has been demonstrated in renal IR injury [8]. Furthermore, the roles of several protective and pathogenic miRNAs have been implicated in AKI and renal fibrosis, which inhibit or promote inflammation, apoptosis and fibrosis [11,12,13,14,15,16]. The role of miR-21 has been most extensively explored in renal IR [17,18,19,20,21,22,23,24,25,26] and fibrosis [27,28,29,30,31,32,33,34,35,36]. MiR-21 expression was elevated after IR and induced tissue protection by promoting angiogenesis [26] and inhibiting inflammation and apoptosis [18,19,22]. However, renal fibrosis sustained the upregulation of miR-21, which contributed to fibrosis progression [33,34]. IR also upregulated miR-146a, which has anti-inflammatory properties and inhibits renal fibrosis and macrophage infiltration [37,38,39]. Nonetheless, the function of most miRNAs implicated in AKI and renal fibrosis has not been thoroughly evaluated, and the results are sometimes conflicting [12,40]. Thus, currently, there is little information available on changes in the renal miRNome following IR-induced fibrosis.

Our aim was to systematically study the time course of the expression pattern of miRNAs after Nx-induced functional recovery in the IR-injured kidney.

## 2. Materials and Methods

### 2.1. Animal Studies

Male Naval Medical Research Institute (NMRI) mice (weighing 25–30 g; Toxi-Coop Ltd., Budapest, Hungary) were used. Mice were housed under standard conditions with free access to food and tap water. In order to reduce the number of experimental animals used, the molecular parameters of the right kidneys were used for control after confirming the functional similarity of right kidneys in mice subjected to IR or sham operation. All protocols were approved by the Pest County Government Office and the Animal Ethics Committee of Semmelweis University (PE/EA/2202-5/2017, date of approval: 14/12/2017). All experiments were performed in accordance with the protocol approved and the relevant guidelines and EU regulations.

### 2.2. Renal Ischemia-Reperfusion and Nephrectomy

Experiments were performed on 104 mice according to the experimental protocol of Skrypnyk et al. [7] as summarized in Table 1. Mice were anesthetized with intraperitoneal (i.p.) injection of ketamine (80 mg/kg; Richter-Gedeon Nyrt., Budapest, Hungary) and xylazine (10 mg/kg; Streuli Pharma AG, Uznach, Switzerland). The body temperature of the animals was kept at 37.0 ± 0.5 °C during the surgeries using a heating pad (Supertech Ltd., Budapest, Hungary). Mice were subjected to either 30 min of renal ischemia/reperfusion (IR) or sham surgery (S) on the left kidney on day 0. Nx or S was performed on day 7, resulting in four groups: IR-S, IR-Nx, S-S and S-Nx. The experiments were terminated 7, 8, 10, 14 or 28 days after the IR surgery. As stated above, the S-S and S-Nx groups were included only in the experiments terminated on days 8 and 10 as we aimed to study the effects of Nx on post-ischemic renal fibrosis progression by comparing the IR-S to the IR-Nx group. Furthermore, all investigated molecular parameters demonstrated strong similarities in the right kidneys in the S-S and S-Nx groups (Supplementary Figure S1).

### 2.3. Organ Harvest

The right kidney was removed either at nephrectomy or at the time of termination. The mice were injected with 5000 U/kg BW heparin i.p. (Ratiopharm GmbH, Ulm, Germany), and 3 min later they were sacrificed by cervical dislocation. The chest was opened and after cross-section of the vena cava, blood was collected from the thoracic cavity. Blood was washed out from the blood vessels by intracardial injection of 10 mL 4 °C saline. The kidneys were removed and decapsulated. One third of the upper pole of the kidneys was placed in 500 µL TRI Reagent (TR 118, Molecular Research Center, Inc., Cincinnati, OH, USA) and was snap frozen in liquid nitrogen and kept at −80 °C for RNA isolation. A 1 mm cross-section of the kidney at the hilus level including all layers of cortex and medulla was fixed in 4% buffered formaldehyde. On the next day, the section was dehydrated and embedded in paraffin (FFPE) for histology. The remaining parts of the kidney were cut into pieces and snap frozen in liquid nitrogen and kept at −80 °C for any further molecular biology analysis.

### 2.4. Plasma Urea Determination

Blood samples were collected on days −1, 1, 6, 8, 9, 10, 14, 21 and 28. The plasma was separated by centrifugation (6000× *g*, 2 min) and stored at −80 °C.

Plasma urea concentrations were measured by a urease and glutamate-dehydrogenase enzymatic assay with colorimetric detection at 340 nm according to the manufacturer’s protocol (Diagnosticum Zrt., Budapest, Hungary). The urea concentration of the samples was determined using a standard curve.

### 2.5. RNA Preparation

Total RNA was extracted from the kidneys with TRI Reagent (TR 118, Molecular Research Center) according to the manufacturer’s protocol. RNA concentration and purity was checked with NanoDrop 2000c spectrophotometer (Thermo Fisher, Waltham, MA, USA), and RNA integrity of the samples was verified by electrophoresis on a 1% agarose gel (Invitrogen Ltd., Paisley, UK). The RNA solutions were stored at −80 °C until further analysis.

### 2.6. qPCR Analysis of Gene Expression

Tumor necrosis factor alpha (Tnf-α; an inflammatory marker) and transforming growth factor beta (Tgf-β; a fibrosis marker) mRNA levels were measured in the kidneys. Renal tubular damage was assessed based on lipocalin-2 (Lcn-2), also called neutrophil gelatinase-associated lipocalin (NGAL) gene expression. 18S rDNA was used as reference gene. Right kidneys served as controls. Messenger RNA (mRNA) expression from kidney tissue homogenates was measured as described previously. Reverse transcription into cDNA was performed using the High-Capacity cDNA Archive Kit (Applied Biosystems, Foster City, CA, USA) according to the protocol provided by the manufacturer. Gene expression was evaluated on the Bio-Rad C1000™ Thermal Cycler with CFX96™ Optics Module real-time PCR system (Bio-Rad Laboratories, Inc., Hercules, California, USA). The qPCR reaction was carried out with SensiFAST™ SYBR No-ROX Kit (Bioline Reagents Limited, London, UK), according to the manufacturer’s protocol. Primer annealing was set to 60 °C. Primers (Table 2) were designed by NCBI/Primer-BLAST online software and synthesized by Integrated DNA Technologies (Integrated DNA Technologies, Inc., Coralville, IA, USA). All samples were measured in duplicates. Gene expression was calculated using the relative quantification (ΔΔC_q_) method. Standard curves were used for verifying the efficiency of the qPCR reaction. The melting curves were also checked for abnormalities of the PCR products.

### 2.7. MicroRNA Microarray of 1195 Targets

Representative left kidney samples from each group of the 8-day experiment (6-6 samples from the IR-S and IR-Nx groups and 4-4 samples from the S-S and S-Nx groups) were selected for miRNA profiling based on plasma urea levels and the Tnf-α and Tgf-β mRNA expressions in such a way that the mean and standard deviation of the original and selected values were similar (the samples represented their groups).

The microarray measurements were carried out by Exiqon A/S (Vedbæk, Denmark). In brief, the quality of the total RNA samples was verified by Agilent 2100 Bioanalyzer (Agilent Technologies Inc., Santa Clara, CA, USA). The reference sample was generated by pooling a fraction of the RNA samples. A measurement of 750 ng total RNA from both the test and reference samples was labelled with fluorescent Hy3 and Hy5 labels using the miRCURY LNA™ microRNA Hi-Power Labeling Kit, Hy3™/Hy5™ (Exiqon, Denmark). Hybridization was performed according to the miRCURY LNA™ microRNA Array instruction manual using a Tecan HS4800™ hybridization station (Tecan, Austria). After hybridization, the slides were scanned using the Agilent G2565BA Microarray Scanner System (Agilent). Image analysis was carried out with ImaGene^®^ 9 (miRCURY LNA™ microRNA Array Analysis Software, Exiqon, Denmark). The quantified signals were background corrected (Normexp with offset value 10) and normalized using the global Lowess (Locally Weighted Scatterplot Smoothing) regression algorithm. Microarray data discussed in this publication have been deposited in NCBI’s Gene Expression Omnibus [41] and are accessible through GEO Series accession number GSE157221 (https://www.ncbi.nlm.nih.gov/geo/query/acc.cgi?acc=GSE157221 (accessed on 1 September 2020)).

### 2.8. MicroRNA qPCR

For validation of the microarray results, miRNA expression was measured in all samples from the 8-day experiment by real-time PCR. Expression of successfully validated miRNAs was measured in all samples from each time point. MiR-2137 was opted out of further measurements because of its low signal intensity in the qPCR amplification. Reverse transcription into cDNA was carried out by the Applied Biosystem™ TaqMan™ Advanced miRNA cDNA Synthesis Kit (Applied Biosystems) according to the manufacturer’s protocol. MicroRNA expression was evaluated using TaqMan™ Advanced miRNA Assays and TaqMan™ Fast Advanced Master Mix (Applied Biosystems). All samples were measured in duplicates and expression was calculated using the relative quantification (ΔΔCq) method. Let-7g-5p was used as reference.

All miRNAs upregulated by IR with significant fold-changes (FCs) above 2.5 were included in the validation except miR-199b-5p, for which TaqMan™ Advanced miRNA Assay was not available. Three other miRNAs upregulated by IR with significant FCs above 1.5 were also selected for validation based on literature data or because they belong to miRNA families or clusters of other validated miRNAs. MicroRNA expression changes were considered verified if they were altered the same way significantly both by the microarray and qPCR.

### 2.9. MicroRNA Target Network Analysis

We performed miRNA target network analysis for those miRNAs that were verified by qPCR. To identify mRNAs likely regulated by differentially expressed miRNAs, we constructed miRNA-target interaction networks as implemented previously in other studies [42,43,44] using the miRNAtarget software (mirnatarget.com (accessed several times in October, 2020), Pharmahungary, Szeged, Hungary). The miRNAtarget software integrates data specific for Mus musculus from the experimentally validated, manually curated miRTarBase [45] database and in silico miRNA-target prediction databases, miRDB [46] and TargetScan [47]. In particular, the following miRNA-target interaction data sources were used: miRDB v5.0 (released in August 2014) filtered for Mus musculus records based on taxonomy ID, ‘Conserved Sites context++ scores’ file from TargetScan Mouse 7.2 (released in August, 2018) in which context++ scores of miRNA binding sites were summed for each transcript, yielding total context++ scores and miRTarBase 8.0. Predictions with miRDB scores and TargetScan total context++ scores ≤80 and ≥−0.2, respectively, were excluded from further analysis. In the resulting networks, miRNAs and mRNAs are represented as nodes, and edges symbolize predicted miRNA-target interactions. To visualize the networks we used EntOptLayout plugin (version 2.1) [48] for Cytoscape (version 3.8.0), alternately applying position and with optimization steps.

### 2.10. Western Blot

Western blot was performed as previously described (Kucsera D. et al. 2021, Front Physiol., PMID: 33692700). Briefly, frozen kidney samples were homogenized in RIPA buffer (Cell Signalling Technology, Danvers, MA, USA). After protein concentration measurement, 20 μg of protein was loaded onto 4–20% polyacrylamide gels for separation with 90 V, and proteins were transferred (Criterion Blotter, BioRad, Hercules, CA, USA) onto PVDF membranes (BioRad, Hercules, CA, USA). After a blocking step with 5% BSA in 0.05% Tris-buffered saline with Tween 20 (TBS-T), the membrane was incubated overnight at 4 °C with the following primary antibodies (dissolved in 5% BSA solution): CD2AP (5478, Cell Signaling Technology, Danvers, MA, USA, 1:1000 dilution) and PLEXIN A2 (6896, Cell Signaling Technology, Danvers, MA, USA, 1:1000 dilution). After washing the membranes with 0.05% TBS-T (3 × 10 min), the membrane was incubated with a secondary antibody (horseradish peroxidase-conjugated goat anti-rabbit, 7074, Cell Signaling Technology, Danvers, MA, USA, 1:2000 dilution) dissolved in 5% BSA solution for 2 h at room temperature, followed by 3 × 10 min wash. For band detection, the membranes were incubated with enhanced chemiluminescence reagent (Clarity Western ECL Substrate or Clarity Max Western, BioRad, Hercules, CA, USA) for 5 min, and the signal was recorded with the ChemiDoc XRS + System (BioRad, Hercules, CA, USA). Band intensity was evaluated using the Image Lab Software (BioRad, Hercules, CA, USA). Loading control was determined by measuring protein content by staining with the MemCode stain (24585, Pierce™ Reversible Protein Stain Kit for PVDF Membranes, Thermo Fisher Scientific, Waltham, MA, USA).

### 2.11. Statistical Analysis

Only miRNAs with an average Hy3 signal intensity above 6.0 were included in the analysis of the miArray after normalization by Lowess regression algorithm (M-value). Relative miRNA expression values were calculated as 2^M-value^. FCs were log2 transformed and compared to the right kidneys using paired *t*-test. Differentially expressed miRNAs were further analysed if *p* < 0.05 and FC ≥ 1.5.

Messenger RNA and miRNA FCs as determined by RT-qPCR were calculated by dividing each normalized expression value with the mean of the respective control group. Outliers were determined with the ROUT method (combination of a robust nonlinear regression and an outlier identification method based on the false discovery rate) [49]. Two-way ANOVA followed by Tukey’s multiple comparisons test was used for between-group comparisons. ANOVA was performed after logarithmic transformation of the data if Bartlett’s test indicated inhomogeneity of variances.

The null hypothesis was rejected if *p* < 0.05. Statistical analysis was performed using GraphPad Prism6 (GraphPad Software Inc., San Diego, CA, USA) and IBM SPSS v25 Software (International Business Machines Corp., Armonk, NY, USA). Continuous data are expressed as mean ± standard error of the mean (SEM) unless otherwise stated.

## 3. Results

### 3.1. Effects of Delayed Contralateral Nephrectomy on Kidney Function

Our previous study demonstrated that Nx performed on day 7 almost fully restored the function of the otherwise hardly functioning and atrophying post-ischemic kidney [9]. IR significantly upregulated the renal expression of genes related to fibrosis, inflammation and oxidative stress in the post-ischemic kidney, while Nx significantly attenuated these changes at all times studied (published in our previous paper [9]).

**Figure 1:**
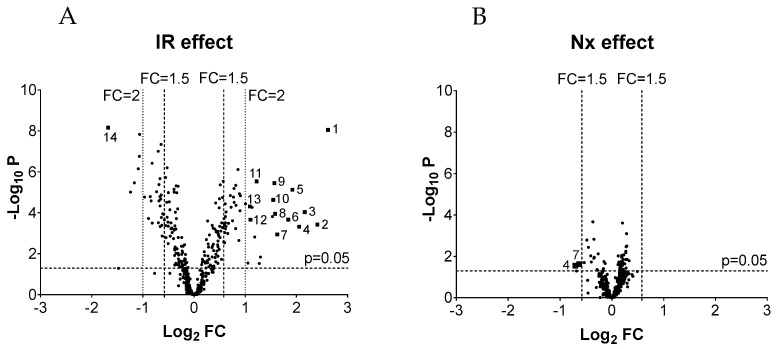
The effects of ischemia-reperfusion (IR) injury and delayed contralateral nephrectomy (Nx) on miRNA expression in the kidneys 8 days after IR and 1 day after Nx based on the miRNA microarray. The level of significance (given as −log_10_ *p* value) is plotted against the fold change (FC, given as log_2_FC). Vertical lines mark 1.5× and 2× FC, while the horizontal line marks the significance level (*p* < 0.05). (**A**): Effect of IR (IR-S vs. S-S). ▪: miRNAs selected for validation by qPCR; 1: mmu-miR-21a-5p, 2: mmu-miR-2137, 3: mmu-miR-142-3p, 4: mmu-miR-762, 5: mmu-miR-223-3p, 6: mmu-miR-142-5p, 7: mmu-miR-2861, 8: mmu-miR-3102-5p, 9: mmu-miR-199a-5p, 10: mmu-miR-199a-3p/mmu-miR-199b-3p, 11: mmu-miR-214-3p, 12: mmu-miR-146a-5p, 13: mmu-miR-21a-3p, 14: mmu-miR-129-1-3p. (**B**): Effects of Nx (IR-Nx vs. IR-S).

IR significantly upregulated the expression of 43 miRNAs at least 1.5-fold and 20 miRNAs at least twofold 8 days after the injury (Figure 1A, Supplementary Table S1). At the same time, 29 miRNAs were significantly downregulated at least 1.5-fold (Figure 1A, Supplementary Table S2), but only six miRNAs were downregulated more than twofold. One day after nephrectomy, only small changes in miRNA expression were observed in the IR-Nx group in comparison to the IR-S group (Figure 1B). Nx did not upregulate any of the miRNAs and downregulated only two miRNAs more than 1.5-fold (miR-762 (FC = 0.61, *p* = 0.0280) and miR-2861 (FC = 0.65, *p* = 0.0237)) in the IR-injured kidneys.

### 3.2. MicroRNA Validation

We attempted to validate all miRNAs up- or downregulated at least 2.5-fold using qPCR (except miR-199b-5p, as no assay was available). Furthermore, we included three more miRNAs from the top 20 upregulated miRNAs for validation (Table 3): miR-214-3p (clustered with miR-199a), miR-21a-3p (miR-21 family member) and miR-146a-5p (known role in AKI [17,37,50]). The two miRNAs downregulated at least 1.5x by Nx (miR-762 and miR-2861) were also included in the validation as they were among the miRNAs most affected by IR.

**Figure 2:**
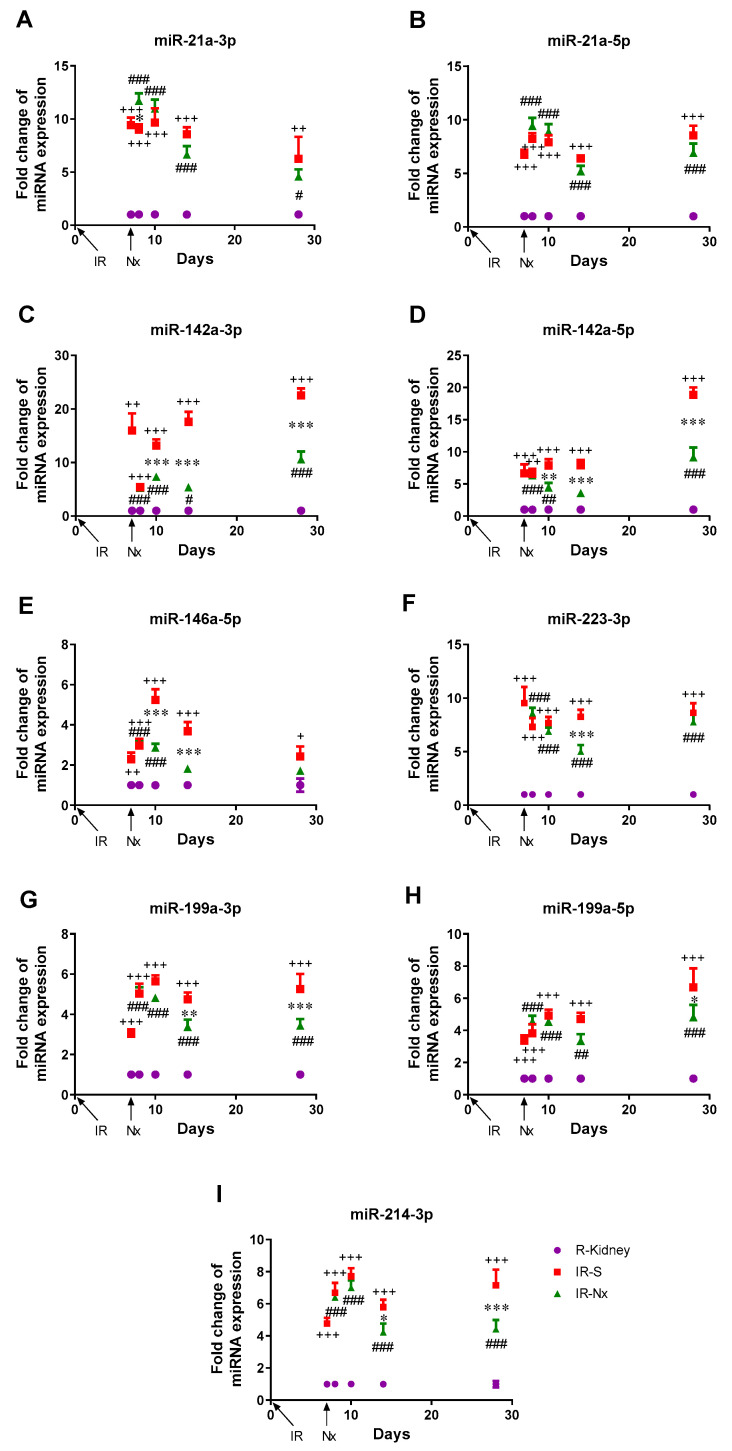
Fold changes of miRNA expression in the kidneys of mice relative to the control right kidneys. The experiments were terminated 7, 8, 10, 14 and 28 days after IR. Red: IR-S left kidney, green: IR-Nx left kidney, purple: control right kidneys. +: IR-S vs. control, #: IR-Nx vs. control, *: IR-Nx vs. IR-S. */#: *p* < 0.05, **/++/##: *p* < 0.01, ***/+++/###: *p* < 0.001. (**A**): miR-21a-3p, (**B**): miR-21a-5p, (**C**): miR-142a-3p, (**D**): miR-142a-5p, (**E**): miR-146a-5p, (**F**): miR-142a-3p, (**G**): miR-223-3p, (**H**): miR-199a-5p, (**I**): miR-214-3p.

Real-time PCR validation verified the results of the microarray in 9 out of 14 miRNAs (Table 3). They were similarly upregulated in the left kidneys in the IR-S and the IR-Nx groups compared to the S-S group (Supplementary Table S3) and also to the contralateral right kidneys (Figure 2). The MRNA expression of Tnf-α, Tgf-β, Lcn-2 and all miRNAs was similar in the left kidneys in the S-S and S-Nx groups and the right kidneys in all groups (Supplementary Table S4).

Real-time PCR validation confirmed that miR-21a-3p expression was upregulated in the IR-injured kidneys (Table S3). Although there was no difference in its expression between the IR-S and IR-Nx left kidneys based on the microarray, we detected significantly elevated miR-21a-3p expression in the IR-Nx group compared to the IR-S group by qPCR (Supplementary Table S3 and Figure 2).

Validation of the expression changes of miR-762, miR-2861, miR-3102-5p and miR-129-1-3p was unsuccessful as we found no significant differences in miR-762, miR-3102-5p and miR-129-1-3p expression either in the IR-injured left kidneys compared to the S-S left kidneys or in the IR-Nx group compared to the IR-S group (Table 3). MiR-2861 expression was below the limit of detection.

### 3.3. Temporal Changes of MicroRNA Expression

We evaluated the expression of nine miRNAs (miR-21a duplex (miR-21a-3p, miR-21a-5p), miR-142a duplex, miR-146a-5p, miR-199a duplex, miR-214-3p and miR-223-3p) in all the samples. IR significantly upregulated all miRNAs at all time points (Figure 2). Nx significantly decreased the expression of seven out of the nine miRNAs at least at one time point. However, miR-21a-3p was increased, but miR-21a-5p was not altered on day 8. Nx significantly reduced the expression of miR-142a-duplex and miR-146a-5p on day 10 and further suppressed the expression of miR-142a-5p and miR-146a-5p on day 14, as their expression did not differ significantly in the IR-Nx and right kidneys. MiR-142a-3p and miR-142a-5p expression remained suppressed on day 28 but stayed significantly upregulated compared to the right kidneys. MiR-223-3p was downregulated by Nx only on day 14. MiR-199a-3p and miR-214-3p expression was reduced on days 14 and 28, while miR-199a-5p was diminished only on day 28.

### 3.4. MicroRNA Target Network Analysis

**Figure 3:**
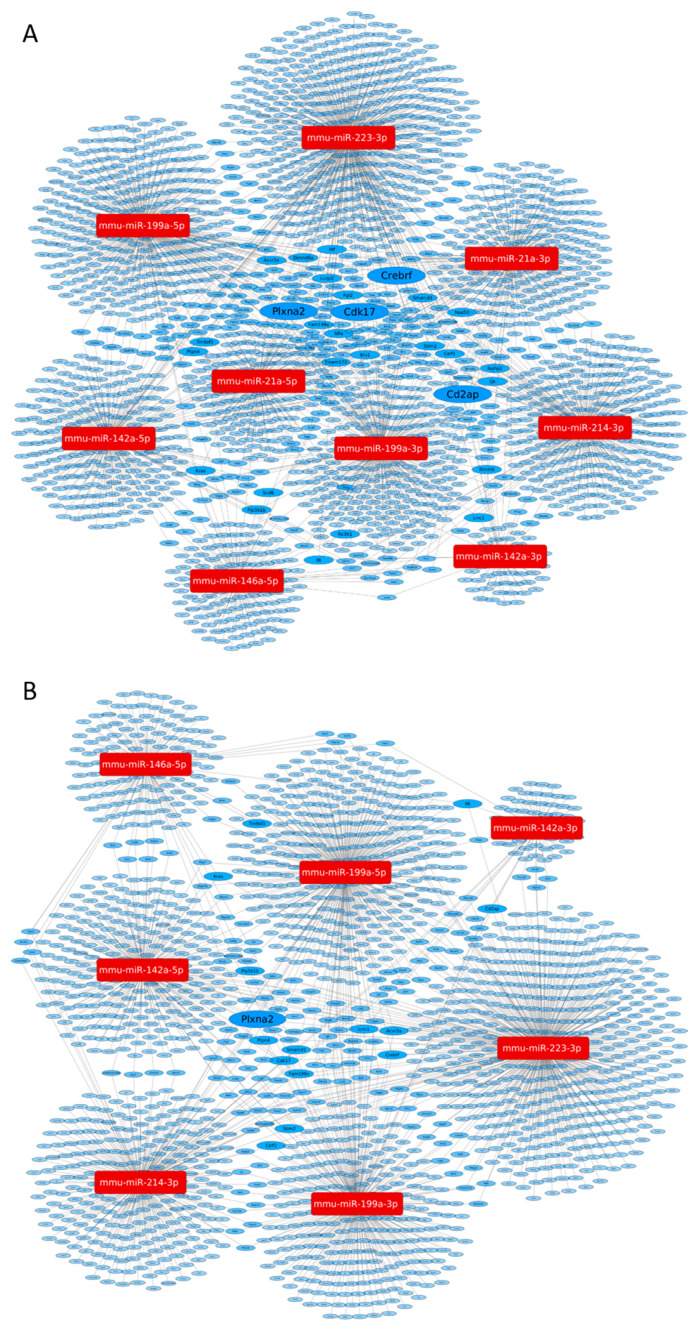
Visual presentation of the miRNA-target network predicted by the miRNAtarget software. (**A**) The effects of ischemia-reprefusion injury. The miRNA-target network was constructed for mmu-miR-21a-duplex, 142a-duplex, 146a-5p, 199a-duplex, 214-3p and 223-3p (red boxes). (**B**) The nephrectomy-target network was constructed for the same miRs, except miR-21a-duplex, as miR-21a-duplex was not modified by nephrectomy. Predicted targets are presented in blue ovals. The size of the hubs are proportional to the number of connections to miRNAs. Predicted associations are listed in Table 4.

The miRNAtarget software for theoretic network analysis of successfully validated miRNAs (Table 4) identified several targets associated with more than one miRNA (Figure 3).

Four genes (CD2-associated protein (CD2AP), cyclin-dependent kinase 17 (Cdk17), CREB3 regulatory factor (Crebrf) and plexin A2 (PlxnA2)) were predicted targets of four miRNAs, and 24 proteins could be associated with three miRNAs (Table 4A). MicroRNAs involved in the regulation of these targets were: miR-21a-3p, miR-142a-duplex, miR-199a-duplex, miR-214-3p and miR-223-3p.

The theoretical network analysis of successfully validated miRNAs that were reduced after Nx revealed only PlxnA2 that was regulated by four miRNAs, and further 14 genes were associated with three miRNAs. As, surprisingly, miR-21 was not influenced by Nx, miR-21 was left out of the analysis. PlxnA2 is a predicted target of miR-142a-5p, miR-199a-5p, miR-214-3p and miR-223-3p.

### 3.5. MicroRNA Target Verification

**Figure 4:**
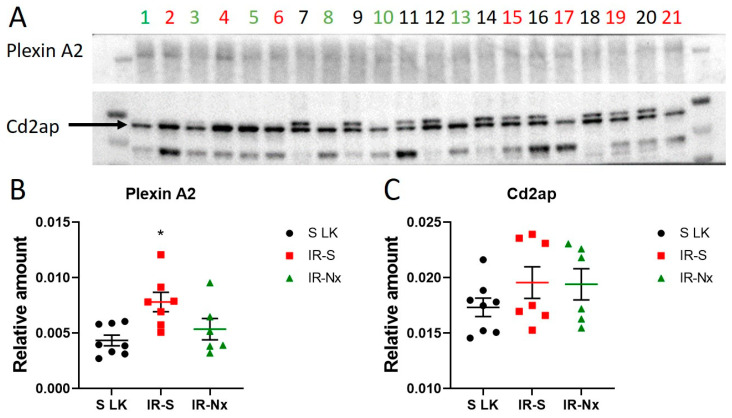
Plexin A2 and Cd2ap protein expression in the kidneys of mice relative to total protein amount 10 days after IR. (**A**) Western blot images of Plexin A2 and Cd2ap. Optical density analysis of images, showing the target protein level relative to total protein amount, (**B**) Plexin A2, (**C**) Cd2ap. Red: IR-S left kidney, green: IR-Nx left kidney, balck: sham left kidneys. One-way ANOVA, *: *p* < 0.05.

To verify the predicted functional relationship between miRNAs and their predicted targets, we measured PlxnA2 and Cd2AP protein expression in the kidneys 10 days after IR (Figure 4). Compared to sham left kidneys, PlxnA2 protein expression was significantly elevated in the IR-S group but not in the IR-Nx group (Figure 4B). Cd2AP protein expression was unchanged in both the IR-S and IR-Nx groups compared to the sham kidneys (Figure 4C).

## 4. Discussion

Unilateral renal ischemia-reperfusion (IR) without nephrectomy (Nx) led to severe and rapidly progressing functional and morphological deterioration of the post-ischemic kidney [8,9]. Nx significantly improved the function of the post-ischemic kidneys and decelerated the progression of fibrosis in the long term, enabling the kidney to function up to 20 weeks after IR [7,9]. The microRNA regulation of the Nx-induced functional recovery is unknown. Therefore, we determined the changes in the expression pattern of miRNAs in the IR-injured kidney after delayed Nx.

Microarray was performed 8 days after IR and 1 day after Nx. We found that IR upregulated and downregulated the renal expression of 43 and 29 miRNAs at least 1.5-fold, respectively. Several miRNAs were altered more than 2–3-fold. However, the Exiqon microarray works with low stringent criteria for detection, potentially resulting in a high rate of false positives [51,52]. Furthermore, when a miRNA has high sequence similarity to other RNA species, they may interfere with the microarray result. Not surprisingly, we failed to validate the expression changes of miRNAs in a few cases (miR-762, miR-2861 and miR-3102-5p).

Our verified results are in line with previous data demonstrating that renal IR upregulated miR-21a-5p [17,20,28], miR-146a [17,28,37], miR-199a-3p [17,28] and miR-214 [17,28,44] for a longer period, from week 1 to week 4, in mice. MiR-142a-3p, miR-199a-5p and miR-223 were shown to be upregulated 10 days after IR [28]. Our study revealed that they were upregulated for a longer period, from day 7 to day 28. In addition, we found that IR also upregulated miR-21a-3p and miR-142a-5p from day 7 to day 28.

We focused on the Nx-induced miRNome changes and, more importantly, aimed to identify changes in the expression of those miRNAs which have a role in fibrosis progression. Generally, Nx inhibited most miRNAs upregulated by IR. Although Tnf-α and Tgf-β mRNA were already downregulated 1 day after Nx, the renal miRNome changes started 3–7 days after Nx. Upregulation of the miR-142a duplex and miR-146a-5p was already reversed 3 days after Nx, followed by a decrease in the expression of miR-199a duplex, miR-214a-3p and miR-223-3p 7 days after Nx.

The most affected miRNA, miR-142a duplex, was markedly downregulated by Nx from day 10 until day 28. Renal expression of miR-142a-5p was also upregulated 10 weeks after the development of hyperglycemia in mice with diabetic nephropathy [53]. Oleanolic acid decreased both fibrosis and miR-142a-5p expression [53]. The above findings support that renal upregulation of miR-142a-5p is deleterious and can be associated with kidney fibrosis. MiR-142a-3p has not yet been studied in renal IR injury. In human kidney transplantation, miR-142-3p was upregulated during allograft rejection [54,55]. MiR-142-3p was induced by TGF-β [56]. Thus, the Nx-induced suppression of the miR-142a duplex might have a role in improved renal function and attenuated fibrogenesis [9].

Nx robustly decreased miR-146a-5p on days 10 and 14, although this effect vanished on day 28. MiR-146a was upregulated from day 7 after unilateral IR [17], and the overexpression of miR-146a at the time of IR protected the kidneys from IR-induced damage [50]. Similarly, the IR-induced renal damage was more severe in miR-146a knockout than in WT mice [37]. The overexpression of miR-146a also reduced the extent of fibrosis and inflammation in the kidneys 6 days after UUO [38]. These results indicate that miR-146a expression is renoprotective at the time of the ischemic insult but may not be advantageous at later stages. In a mouse model of spontaneous chronic renal inflammation, miR-146a expression was elevated especially around the interstitial lesions [57]. Thus, Nx-induced downregulation of miR-146a may be one factor responsible for the developing fibrosis.

In our study, Nx diminished miR-199a-3p and miR-214-3p expression on days 14 and 28, while miR-199a-5p was downregulated only on day 28. MiR-199a-3p delivery to the kidney inhibited the IR-induced apoptosis [58]. MiR-199a-3p was also upregulated after 5/6 nephrectomy [59]. MiR-199a-5p has been investigated more extensively. At the very early stages, miR-199a-5p protected the kidneys against IR-induced damage by suppressing endoplasmic reticular stress [60]. However, miR-199a-5p promoted fibrosis and inflammation at the same time. MiR-199a-5p mediated the TGF-β-induced fibrogenesis [61,62], and its expression correlated with elevated fibrosis markers (e.g., fibronectin) and immune cell chemoattractant levels [63] and enhanced the activity of the TLR4/NF-kB signalling pathway [63]. Considering the pro-fibrotic and pro-inflammatory functions of the miR-199a duplex, lower miR-199a expression after Nx could be beneficial and probably contributed to suppressing inflammation and slowing fibrosis progression in the IR-injured kidneys.

MiR-214 upregulation improved kidney function during the first 72 h after IR by suppressing apoptosis, while miR-214 inhibition had the opposite effect [64]. On the other hand, both knockout and knockdown of miR-214 attenuated UUO-induced renal fibrosis 7 and 14 days after surgery [65,66]. Proximal tubule-specific knockout of miR-214 also decreased fibrosis after both UUO and IR [67]. These results indicate that while shortly after IR, miR-214 limited the IR-induced renal damage, partial or total absence of miR-214 reduced the extent of fibrosis 1–2 weeks post-injury. Thus, the Nx-induced suppression of miR-214 may have contributed to slowing the rate of fibrosis in our study.

We have found that Nx suppressed the IR-induced upregulation of miR-223-3p on day 14 only. The role of miR-223-3p in renal IR and fibrosis has not yet been thoroughly investigated. MiR-223-3p contributed to the protective effects of mesenchymal stem cell delivery against the IR-induced early renal damage [68,69] and suppressed the inflammasome in renal tubular epithelial cells [68]. On the other hand, miR-223-3p was found to promote cardiac fibrosis [70,71]. Considering the pro-fibrotic characteristics of miR-223-3p, its Nx-induced downregulation might have decreased the rate of fibrosis.

Surprisingly, the expression of miR-21a-3p or -5p was hardly influenced despite the fact that they have well-documented pro-fibrotic properties in the kidney [28,33,34]. A possible explanation as to why miR-21 was not reduced by Nx is that upon induction by Tgf-β, miR-21 upregulation is maintained through an autoregulatory feedback loop in fibroblasts, driving the progression of fibrosis [28,29] independently from Tgf-β [34]. Our study demonstrated that Nx decreased renal TGF-β expression, but miR-21a-5p remained upregulated. Therefore, elevated miR-21 expression was one of the driving forces of fibrosis progression in our model.

The miRNA target network analysis of ischemia-regulated miRNAs revealed four genes associated with four of the studied miRNAs. CD2AP has been studied the most in renal pathophysiology and IR. CD2AP is expressed in the whole nephron (glomerulus, proximal and distal tubules and collecting duct) and is a predicted target of fibromirs miRs-21a-3p, -142a-3p, -199a-3p and -223-3p, which were upregulated by ischemia in our study. CD2AP has a role in glomerular filtration by maintaining podocyte intercellular junctions, and its deficiency leads to albumin excretion and nephrotic syndrome [72,73,74]. In a similar study where Nx was performed 8 days after IR, both mRNA and protein expression of CD2AP was downregulated in mouse kidneys on day 28 and also in podocytes 3 weeks after Nx [75]. However, in our study, renal CD2AP protein expression was unchanged 10 days after IR and 3 days after Nx. Therefore, it can be hypothesized that CD2AP is downregulated only at a later time point.

The only protein regulated by four miRNAs in the nephrectomy network (Nx-miRNAs) was PlxnA2. Plexins are receptors for semaphorins [76] in podocytes and regulate the expression of slit-diaphragm proteins and podocyte survival. Semaphorin-plexin signalling has already been shown to be dysregulated in IR in mice, impairing the integrity of the slit diaphragm [74,77]. Additionally, an RNA-Seq study demonstrated the downregulation of KRAS and another isoform of plexin (PlxnB1) in the human kidney after IR injury [78]. Despite the upregulated miRNAs in our study, PlxnA2 was not suppressed 10 days after IR, but it was upregulated. Additionally, following Nx, PlxnA2 showed a decreasing tendency compared to the IR-S group along with the suppression of miRNAs. In a previous study, mRNA expression of PlxnA2 was unchanged 24 h after bilateral IR [77]. Our study shows that PlxnA2 expression is altered later after IR. However, these results are at variance with those predicted. We hypothesize that PlxnA2 expression is more robustly regulated at the promoter level and is only fine-tuned by miRNAs. Day 10 was chosen for Western blot analysis because this is the time when glomerular filtration started to recover rapidly. However, it can also be considered that the effects of miRNAs on protein expression of PlxnA2 and Cd2AP could be demonstrated on days 14 or 28, which possibly warrants further measurements to be performed later.

In conclusion, delayed Nx had a significant impact on the expression of several miRNAs. The diminished expression of the miR-142a-duplex, miR-146a-5p, the miR-199a-duplex, miR-214 and miR-223-3p after delayed nephrectomy could possibly contribute to functional improvement and delayed progression of kidney fibrosis. Despite both PlxnA2 and Cd2AP being predicted targets of several miRNAs, their regulation seems to be dominated by transcriptional factors. However, miRNAs may play a role in the fine-tuning of their expression during renal ischemia-induced fibrosis.

## Figures and Tables

**Table 1:** Summary of the animal experiments performed.

	Number of Animals			
Day of Termination	IR-S Group	IR-Nx Group	S-S Group	S-Nx Group
Day 7	8	-	-	-
Day 8	9	10	6	6
Day 10	9	9	8	7
Day 14	8	7	-	-
Day 28	9	8	-	-

**Table 2:** Sequences of primers used for measuring the expression of target genes by qPCR.

Target	Forward Primer	Reverse Primer
Lcn-2	AGGTGGTACGTTGTGGGC	CTGTACCTGAGGATACCTGTG
Tnf-α	AAATGGCCTCCCTCTCATCA	AGATAGCAAATCGGCTGACG
Tgf-β	CAACAATTCCTGGCGTTACCTTGG	GAAAGCCCTGTATTCCGTCTCCTT
18S	CCAGAATGAGGATCCCAGAA	ACCACCTGAAACATGCAACA

**Table 3:** Validation of the miRNA microarray. One-way ANOVA with S-S, IR-S and IR-Nx groups.

	miRNA	Fold Change (S-S vs. IR-S, Microarray)	*p* Value ANOVA (Microarray)	Fold Change (S-S vs. IR-S, qPCR)	*p* Value ANOVA (qPCR)	Result of Validation
		miRNAs upregulated by IR				
1	mmu-miR-21a-5p	6.13 ± 0.63	0.0000	8.09 ± 1.35	0.000	verified
2	mmu-miR-2137	5.31 ± 1.88	0.0004	4.27 ± 2.51	0.011	verified
3	mmu-miR-142-3p	4.48 ± 1.31	0.0001	7.38 ± 2.10	0.000	verified
4	mmu-miR-762	4.15 ± 1.46	0.0005	0.96 ± 0.36	0.900	failed
5	mmu-miR-223-3p	3.79 ± 0.79	0.0000	5.42 ± 1.65	0.000	verified
6	mmu-miR-142-5p	3.58 ± 0.81	0.0002	9.63 ± 3.08	0.000	verified
7	mmu-miR-2861	3.08 ± 0.94	0.0011	-	-	failed
8	mmu-miR-3102-5p	2.99 ± 0.77	0.0001	0.64 ± 0.23	0.136	failed
9	mmu-miR-199a-5p	2.97 ± 0.39	0.0000	3.45 ± 1.34	0.000	verified
10	mmu-miR-199a-3p/ mmu-miR-199b-3p	2.92 ± 0.53	0.0000	4.03 ± 1.05	0.000	verified
11	mmu-miR-214-3p	2.33 ± 0.16	0.0000	4.19 ± 1.01	0.000	verified
12	mmu-miR-146a-5p	2.15 ± 0.45	0.0002	2.46 ± 0.75	0.000	verified
13	mmu-miR-21a-3p	2.13 ± 0.36	0.0000	12.91 ± 1.98	0.000	partially verified
		miRNAs downregulated by IR				
1	mmu-miR-129-1-3p	0.31 ± 0.06	0.0000	0.33 ± 0.19	0.174	failed

**Table 4:** Predicted target genes of miRNAs in the miRNA-target network built by the miRNAtarget software. In the miRTarBase 8.0 column ‘+’ denotes that experimental evidence is available for the miRNA-target interaction in miRTarBase 8.0 database. Target-prediction algorithm scores (miRDB v5.0 score and TargetScan Mouse 7.2 context++ score) for each miRNA are listed if the corresponding threshold criterion (>80 and <−0.2, respectively) is met, otherwise a ‘−’ symbol is shown. (**A**) The effects of ischemia-reprefusion injury. The miRNA-target network was constructed for mmu-miR-21a-duplex, 142a-duplex, 146a-5p, -199a-duplex, 214-3p and 223-3p. (**B**) The nephrectomy network was constructed for the same miRs, except miR-21a-duplex, as miR-21a-duplex was not regulated by nephroctomy.

A	Gene Symbol	NCBI Gene Description	miRNA Target Strength	Associated miRNAs	miRTarBase 8.0	miRDB v5.0 Score	TargetScan Mouse 7.2 Context++ Score
1	CD2AP	CD2-associated protein	4	mmu-miR-21a-3p	−	93.3	-
				mmu-miR-142a-3p	+	-	-
				mmu-miR-199a-3p	−	94.5	−0.603
				mmu-miR-223-3p	−	-	−0.299
2	Cdk17	cyclin-dependent kinase 17	4	mmu-miR-21a-3p	−	95.4	−0.210
				mmu-miR-142a-5p	−	93.2	-
				mmu-miR-199a-3p	−	88.3	−0.342
				mmu-miR-223-3p	−	98.8	−0.834
3	Crebrf	CREB3 regulatory factor	4	mmu-miR-21a-3p	−	90.4	-
				mmu-miR-199a-3p	−	-	−0.220
				mmu-miR-199a-5p	−	-	−0.213
				mmu-miR-223-3p	−	80.4	-
4	PlxnA2	plexin A2	4	mmu-miR-142a-5p	−	91.1	-
				mmu-miR-199a-5p	−	95.5	−0.369
				mmu-miR-214-3p	−	82.4	-
				mmu-miR-223-3p	+	-	-
5	Acvr2a	activin receptor IIA	3	mmu-miR-199a-3p	−	98.6	−0.614
				mmu-miR-199a-5p	−	-	−0.306
				mmu-miR-223-3p	−	94.2	−0.436
6	Celf1	CUGBP, Elav-like family member 1	3	mmu-miR-199a-3p	−	-	−0.253
				mmu-miR-214-3p	−	81.0	-
				mmu-miR-223-3p	+	-	-
7	Dennd6a	DENN/MADD domain containing 6A	3	mmu-miR-21a-3p	−	94.6	-
				mmu-miR-199a-5p	−	-	−0.236
				mmu-miR-223-3p	+	-	-
8	Etv1	ets variant 1	3	mmu-miR-21a-3p	−	99.5	-
				mmu-miR-21a-5p	−	-	−0.238
				mmu-miR-142a-5p	−	92.8	-
9	Fam199x	family with sequence similarity 199, X-linked	3	mmu-miR-142a-5p	−	84.4	-
				mmu-miR-199a-3p	−	96.0	−0.485
				mmu-miR-223-3p	−	93.5	-
10	Fgl2	fibrinogen-like protein 2	3	mmu-miR-21a-3p	−	93.8	-
				mmu-miR-199a-3p	−	-	−0.208
				mmu-miR-199a-5p	+	-	-
11	Hlf	hepatic leukemia factor	3	mmu-miR-21a-3p	−	99.1	-
				mmu-miR-199a-5p	−	-	−0.299
				mmu-miR-223-3p	+	-	−0.584
12	Il6	interleukin 6	3	mmu-miR-142a-3p	+	-	-
				mmu-miR-146a-5p	+	-	-
				mmu-miR-223-3p	+	-	-
13	Kras	Kirsten rat sarcoma viral oncogene homolog	3	mmu-miR-142a-5p	−	92.5	-
				mmu-miR-146a-5p	−	88.6	-
				mmu-miR-223-3p	+	-	-
14	Lrrc1	leucine rich repeat containing 1	3	mmu-miR-142a-3p	−	92.9	-
				mmu-miR-199a-3p	−	-	−0.272
				mmu-miR-214-3p	−	81.7	-
15	Luzp2	leucine zipper protein 2	3	mmu-miR-21a-3p	−	97.1	-
				mmu-miR-142a-5p	−	-	−0.205
				mmu-miR-223-3p	+	-	-
16	Naa50	N(alpha)-acetyltransferase 50, NatE catalytic subunit	3	mmu-miR-21a-3p	−	99.7	-
				mmu-miR-214-3p	−	94.6	-
				mmu-miR-223-3p	−	86.6	−0.284
17	Nfia	nuclear factor I/A	3	mmu-miR-21a-5p	−	-	−0.321
				mmu-miR-199a-3p	−	-	−0.268
				mmu-miR-223-3p	−	-	−0.296
18	Nufip2	nuclear fragile X mental retardation protein interacting protein 2	3	mmu-miR-21a-3p	−	83.0	-
				mmu-miR-199a-3p	−	88.6	−0.330
				mmu-miR-214-3p	−	98.7	-
19	Pip5k1b	phosphatidylinositol-4-phosphate 5-kinase, type 1 beta	3	mmu-miR-142a-5p	−	-	−0.202
				mmu-miR-146a-5p	−	-	−0.236
				mmu-miR-199a-3p	−	88.7	−0.319
20	Ptpn4	protein tyrosine phosphatase, non-receptor type 4	3	mmu-miR-142a-5p	−	97.9	-
				mmu-miR-199a-3p	−	-	−0.274
				mmu-miR-199a-5p	−	-	−0.253
21	Qk	quaking	3	mmu-miR-21a-3p	−	84.5	-
				mmu-miR-199a-3p	+	-	-
				mmu-miR-214-3p	−	82.9	-
22	Rc3h1	RING CCCH (C3H) domains 1	3	mmu-miR-21a-5p	+	-	-
				mmu-miR-146a-5p	+	-	-
				mmu-miR-214-3p	−	86.3	-
23	Rimklb	ribosomal modification protein rimK-like family member B	3	mmu-miR-21a-3p	−	98.7	-
				mmu-miR-142a-3p	−	85.7	-
				mmu-miR-199a-3p	−	95.1	-
24	Smarcd1	SWI/SNF related, matrix associated, actin dependent regulator of chromatin, subfamily d, member 1	3	mmu-miR-199a-5p	−	-	−0.238
				mmu-miR-214-3p	−	99.1	-
				mmu-miR-223-3p	−	-	−0.258
25	Srsf6	serine and arginine-rich splicing factor 6	3	mmu-miR-21a-3p	−	83.4	−0.287
				mmu-miR-142a-5p	−	98.7	-
				mmu-miR-146a-5p	−	88.2	-
26	Stim2	stromal interaction molecule 2	3	mmu-miR-199a-3p	−	-	−0.288
				mmu-miR-214-3p	−	80.1	-
				mmu-miR-223-3p	−	95.0	-
27	Tm9sf3	transmembrane 9 superfamily member 3	3	mmu-miR-146a-5p	−	85.4	-
				mmu-miR-199a-5p	−	-	−0.256
				mmu-miR-223-3p	+	-	-
28	Tmem170	transmembrane protein 170	3	mmu-miR-21a-5p	−	-	−0.492
				mmu-miR-199a-3p	−	-	−0.293
				mmu-miR-223-3p	−	-	−0.693
**B**	**Gene Symbol**	**NCBI Gene Description**	**miRNA Target Strength**	**Associated miRNAs**	**miRTarBase 8.0**	**miRDB v5.0 Score**	**TargetScan Mouse 7.2 Context++ Score**
1	PlxnA2	plexin A2	4	mmu-miR-142a-5p	−	91.1	-
				mmu-miR-199a-5p	−	95.5	−0.369
				mmu-miR-214-3p	−	82.4	-
				mmu-miR-223-3p	+	-	-
2	Acvr2a	activin receptor IIA	3	mmu-miR-199a-3p	−	98.6	−0.614
				mmu-miR-199a-5p	−	-	−0.306
				mmu-miR-223-3p	−	94.2	−0.436
3	CD2AP	CD2-associated protein	3	mmu-miR-142a-3p	+	-	-
				mmu-miR-199a-3p	−	94.5	−0.603
				mmu-miR-223-3p	−	-	−0.299
4	Cdk17	cyclin-dependent kinase 17	3	mmu-miR-142a-5p	−	93.2	-
				mmu-miR-199a-3p	−	88.3	−0.342
				mmu-miR-223-3p	−	98.8	−0.834
5	Celf1	CUGBP, Elav-like family member 1	3	mmu-miR-199a-3p	−	-	−0.253
				mmu-miR-214-3p	−	81.0	-
				mmu-miR-223-3p	+	-	-
6	Crebrf	CREB3 regulatory factor	3	mmu-miR-199a-3p	−	-	−0.220
				mmu-miR-199a-5p	−	-	−0.213
				mmu-miR-223-3p	−	80.4	-
7	Fam199x	family with sequence similarity 199, X-linked	3	mmu-miR-142a-5p	−	84.4	-
				mmu-miR-199a-3p	−	96.0	−0.485
				mmu-miR-223-3p	−	93.5	-
8	Il6	interleukin 6	3	mmu-miR-142a-3p	+	-	-
				mmu-miR-146a-5p	+	-	-
				mmu-miR-223-3p	+	-	-
9	Kras	Kirsten rat sarcoma viral oncogene homolog	3	mmu-miR-142a-5p	−	92.5	-
				mmu-miR-146a-5p	−	88.6	-
				mmu-miR-223-3p	+	-	-
10	Lrrc1	leucine rich repeat containing 1	3	mmu-miR-142a-3p	−	92.9	-
				mmu-miR-199a-3p	−	-	−0.272
				mmu-miR-214-3p	−	81.7	-
11	Pip5k1b	phosphatidylinositol-4-phosphate 5-kinase, type 1 beta	3	mmu-miR-142a-5p	−	-	−0.202
				mmu-miR-146a-5p	−	-	−0.236
				mmu-miR-199a-3p	−	88.7	−0.319
12	Ptpn4	protein tyrosine phosphatase, non-receptor type 4	3	mmu-miR-142a-5p	−	97.9	-
				mmu-miR-199a-3p	−	-	−0.274
				mmu-miR-199a-5p	−	-	−0.253
13	Smarcd1	SWI/SNF related, matrix associated, actin dependent regulator of chromatin, subfamily d, member 1	3	mmu-miR-199a-5p	−	-	−0.238
				mmu-miR-214-3p	−	99.1	-
				mmu-miR-223-3p	−	-	−0.258
14	Stim2	stromal interaction molecule 2	3	mmu-miR-199a-3p	−	-	−0.288
				mmu-miR-214-3p	−	80.1	-
				mmu-miR-223-3p	−	95.0	-
15	Tm9sf3	transmembrane 9 superfamily member 3	3	mmu-miR-146a-5p	−	85.4	-
				mmu-miR-199a-5p	−	-	−0.256
				mmu-miR-223-3p	+	-	-

## Data Availability

Microarray data been deposited in NCBI’s Gene Expression Omnibus and are accessible through GEO Series accession number GSE157221 (https://www.ncbi.nlm.nih.gov/geo/query/acc.cgi?acc=GSE157221, accessed on 1 September 2020). All other data are available from the corresponding author upon request.

## Supplementary Materials

Supplementary Materials can be found at https://www.mdpi.com/article/10.3390/biomedicines9070815/s1.

## References

[1] Gonsalez S.R., Cortês A.L., da Silva R.C., Lowe J., Prieto M.C., da Silva Lara L. (2019). Acute kidney injury overview: From basic findings to new prevention and therapy strategies. Pharmacol. Ther..

[2] Liu B.C., Tang T.T., Lv L.L., Lan H.Y. (2018). Renal tubule injury: A driving force toward chronic kidney disease. Kidney Int..

[3] Lee H.J., Feliers D., Barnes J.L., Oh S., Choudhury G.G., Diaz V., Galvan V., Strong R., Nelson J., Salmon A. (2018). Hydrogen sulfide ameliorates aging-associated changes in the kidney. GeroScience.

[4] Hamar P., Kerjaschki D. (2016). Blood capillary rarefaction and lymphatic capillary neoangiogenesis are key contributors to renal allograft fibrosis in an ACE inhibition rat model. Am. J. Physiol. Heart Circ. Physiol..

[5] Nemeth Z., Kokeny G., Godo M., Mózes M., Rosivall L., Gross M.-L., Ritz E., Hamar P. (2009). Increased renoprotection with ACE inhibitor plus aldosterone antagonist as compared to monotherapies--the effect on podocytes. Nephrol. Dial. Transpl..

[6] Joshi S., Chittimalli K., Jahan J., Vasam G., Jarajapu Y.P. (2021). ACE2/ACE imbalance and impaired vasoreparative functions of stem/progenitor cells in aging. GeroScience.

[7] Skrypnyk N.I., Harris R.C., de Caestecker M.P. (2013). Ischemia-reperfusion model of acute kidney injury and post injury fibrosis in mice. J. Vis. Exp..

[8] Zager R.A., Johnson A.C.M., Becker K. (2011). Acute unilateral ischemic renal injury induces progressive renal inflammation, lipid accumulation, histone modification, and “end-stage” kidney disease. Am. J. Physiol. Physiol..

[9] Tod P., Bukosza E.N., Róka B., Kaucsár T., Fintha A., Krenács T., Szénási G., Hamar P. (2020). Post-Ischemic Renal Fibrosis Progression Is Halted by Delayed Contralateral Nephrectomy: The Involvement of Macrophage Activation. Int. J. Mol. Sci..

[10] Kierulf-Lassen C., Nielsen P.M., Qi H., Damgaard M., Laustsen C., Pedersen M., Krag S., Birn H., Nørregaard R., Jespersen B. (2017). Unilateral nephrectomy diminishes ischemic acute kidney injury through enhanced perfusion and reduced pro-inflammatory and pro-fibrotic responses. PLoS ONE.

[11] Zhou P., Chen Z., Zou Y., Wan X. (2016). Roles of Non-Coding RNAs in Acute Kidney Injury. Kidney Blood Press. Res..

[12] Fan P.-C., Chen C.-C., Chen Y.-C., Chang Y.-S., Chu P.-H. (2016). MicroRNAs in acute kidney injury. Hum. Genom..

[13] Banaei S. (2015). Novel role of microRNAs in renal ischemia reperfusion injury. Ren. Fail..

[14] Gholaminejad A., Abdul Tehrani H., Gholami Fesharaki M. (2018). Identification of candidate microRNA biomarkers in renal fibrosis: A meta-analysis of profiling studies. Biomarkers.

[15] Chung A.C.-K., Lan H.Y. (2015). MicroRNAs in renal fibrosis. Front. Physiol..

[16] Kiss T., Giles C.B., Tarantini S., Yabluchanskiy A., Balasubramanian P., Gautam T., Csipo T., Nyúl-Tóth Á., Lipecz A., Szabo C. (2019). Nicotinamide mononucleotide (NMN) supplementation promotes anti-aging miRNA expression profile in the aorta of aged mice, predicting epigenetic rejuvenation and anti-atherogenic effects. GeroScience.

[17] Godwin J.G., Ge X., Stephan K., Jurisch A., Tullius S.G., Iacomini J. (2010). Identification of a microRNA signature of renal ischemia reperfusion injury. Proc. Natl. Acad. Sci. USA.

[18] Hu H., Jiang W., Xi X., Zou C., Ye Z. (2014). MicroRNA-21 attenuates renal ischemia reperfusion injury via targeting caspase signaling in mice. Am. J. Nephrol..

[19] Li Z., Deng X., Kang Z., Wang Y., Xia T., Ding N., Yin Y. (2016). Elevation of miR-21, through targeting MKK3, may be involved in ischemia pretreatment protection from ischemia–reperfusion induced kidney injury. J. Nephrol..

[20] Kaucsár T., Révész C., Godó M., Krenács T., Albert M., Szalay C.I., Rosivall L., Benyó Z., Bátkai S., Thum T. (2013). Activation of the miR-17 Family and miR-21 During Murine Kidney Ischemia-Reperfusion Injury. Nucleic Acid Ther..

[21] Song N., Zhang T., Xu X.L., Lu Z., Yu X., Fang Y., Hu J., Jia P., Teng J., Ding X. (2018). miR-21 protects against ischemia/reperfusion-induced acute kidney injury by preventing epithelial cell apoptosis and inhibiting dendritic cell maturation. Front. Physiol..

[22] Xu X., Kriegel A.J., Liu Y., Usa K., Mladinov D., Liu H., Fang Y., Ding X., Liang M. (2012). Delayed ischemic preconditioning contributes to renal protection by upregulation of miR-21. Kidney Int..

[23] Saikumar J., Hoffmann D., Kim T.-M., Gonzalez V.R., Zhang Q., Goering P.L., Brown R.P., Bijol V., Park P.J., Waikar S.S. (2012). Expression, circulation, and excretion profile of microRNA-21, -155, and -18a following acute kidney injury. Toxicol. Sci..

[24] Pellegrini K.L., Gerlach C.V., Craciun F.L., Ramachandran K., Bijol V., Kissick H.T., Vaidya V.S. (2016). Application of small RNA sequencing to identify microRNAs in acute kidney injury and fibrosis. Toxicol. Appl. Pharmacol..

[25] Liu X., Hong Q., Wang Z., Yu Y., Zou X., Xu L. (2015). MiR-21 inhibits autophagy by targeting Rab11a in renal ischemia/reperfusion. Exp. Cell Res..

[26] Xu X., Song N., Zhang X., Jiao X., Hu J., Liang M., Teng J., Ding X. (2017). Renal Protection Mediated by Hypoxia Inducible Factor-1α Depends on Proangiogenesis Function of miR-21 by Targeting Thrombospondin 1. Transplantation.

[27] Zarjou A., Yang S., Abraham E., Agarwal A., Liu G. (2011). Identification of a microRNA signature in renal fibrosis: Role of miR-21. Am. J. Physiol. Renal Physiol..

[28] Chau B.N., Xin C., Hartner J., Ren S., Castano A.P., Linn G., Li J., Tran P.T., Kaimal V., Huang X. (2012). MicroRNA-21 promotes fibrosis of the kidney by silencing metabolic pathways. Sci. Transl. Med..

[29] Zheng S.-B., Zheng Y., Jin L.-W., Zhou Z.-H., Li Z.-Y. (2018). Microvesicles containing microRNA-21 secreted by proximal tubular epithelial cells are involved in renal interstitial fibrosis by activating AKT pathway. Eur. Rev. Med. Pharmacol. Sci..

[30] Liu X.J., Hong Q., Wang Z., Yu Y.-Y., Zou X., Xu L.-H. (2016). MicroRNA21 promotes interstitial fibrosis via targeting DDAH1: A potential role in renal fibrosis. Mol. Cell. Biochem..

[31] Liu X., Hong Q., Wang Z., Yu Y., Zou X., Xu L. (2016). Transforming growth factor-β-sphingosine kinase 1/S1P signaling upregulates microRNA-21 to promote fibrosis in renal tubular epithelial cells. Exp. Biol. Med..

[32] Chung A.C.K., Dong Y., Yang W., Zhong X., Li R., Lan H.Y. (2013). Smad7 suppresses renal fibrosis via altering expression of TGF-β/Smad3-regulated microRNAs. Mol. Ther..

[33] Zhong X., Chung A.C.K., Chen H.-Y., Meng X.-M., Lan H.Y. (2011). Smad3-mediated upregulation of miR-21 promotes renal fibrosis. J. Am. Soc. Nephrol..

[34] Sun Q., Miao J., Luo J., Yuan Q., Cao H., Su W., Zhou Y., Jiang L., Fang L., Dai C. (2018). The feedback loop between miR-21, PDCD4 and AP-1 functions as a driving force for renal fibrogenesis. J. Cell Sci..

[35] Denby L., Ramdas V., McBride M.W., Wang J., Robinson H., McClure J., Crawford W., Lu R., Hillyard D.Z., Khanin R. (2011). miR-21 and miR-214 are consistently modulated during renal injury in rodent models. Am. J. Pathol..

[36] Tang C.-R., Luo S.-G., Lin X., Wang J., Liu Y. (2019). Silenced miR-21 inhibits renal interstitial fibrosis via targeting ERK1/2 signaling pathway in mice. Eur. Rev. Med. Pharmacol. Sci..

[37] Amrouche L., Desbuissons G., Rabant M., Sauvaget V., Nguyen C., Benon A., Barre P., Rabaté C., Lebreton X., Gallazzini M. (2017). MicroRNA-146a in Human and Experimental Ischemic AKI: CXCL8-Dependent Mechanism of Action. J. Am. Soc. Nephrol..

[38] Morishita Y., Imai T., Yoshizawa H., Watanabe M., Ishibashi K., Muto S., Nagata D. (2015). Delivery of microRNA-146a with polyethylenimine nanoparticles inhibits renal fibrosis in vivo. Int. J. Nanomed..

[39] Huang Y., Wang H., Wang Y., Peng X., Li J., Gu W., He T., Chen M. (2018). Regulation and mechanism of miR-146 on renal ischemia reperfusion injury. Pharmazie.

[40] Lv W., Fan F., Wang Y., Gonzalez-Fernandez E., Wang C., Yang L., Booz G.W., Roman R.J. (2018). Therapeutic potential of microRNAs for the treatment of renal fibrosis and CKD. Physiol. Genom..

[41] Edgar R., Domrachev M., Lash A.E. (2002). Gene Expression Omnibus: NCBI gene expression and hybridization array data repository. Nucleic Acids Res..

[42] Ágg B., Baranyai T., Makkos A., Vető B., Faragó N., Zvara Á., Giricz Z., Veres D.V., Csermely P., Arányi T. (2018). MicroRNA interactome analysis predicts post-transcriptional regulation of ADRB2 and PPP3R1 in the hypercholesterolemic myocardium. Sci. Rep..

[43] Bencsik P., Kiss K., Ágg B., Baán J.A., Ágoston G., Varga A., Gömöri K., Mendler L., Faragó N., Zvara Á. (2019). Sensory Neuropathy Affects Cardiac miRNA Expression Network Targeting IGF-1, SLC2a-12, EIF-4e, and ULK-2 mRNAs. Int. J. Mol. Sci..

[44] Sághy É., Vörös I., Ágg B., Kiss B., Koncsos G., Varga Z.V., Görbe A., Giricz Z., Schulz R., Ferdinandy P. (2020). Cardiac miRNA Expression and their mRNA Targets in a Rat Model of Prediabetes. Int. J. Mol. Sci..

[45] Chou C.-H., Shrestha S., Yang C.-D., Chang N.-W., Lin Y.-L., Liao K.-W., Huang W.-C., Sun T.-H., Tu S.-J., Lee W.-H. (2018). miRTarBase update 2018: A resource for experimentally validated microRNA-target interactions. Nucleic Acids Res..

[46] Chen Y., Wang X. (2020). miRDB: An online database for prediction of functional microRNA targets. Nucleic Acids Res..

[47] Agarwal V., Bell G.W., Nam J.-W., Bartel D.P. (2015). Predicting effective microRNA target sites in mammalian mRNAs. Elife.

[48] Ágg B., Császár A., Szalay-Bekő M., Veres D.V., Mizsei R., Ferdinandy P., Csermely P., Kovács I.A. (2019). The EntOptLayout Cytoscape plug-in for the efficient visualization of major protein complexes in protein-protein interaction and signalling networks. Bioinformatics.

[49] Motulsky H.J., Brown R.E. (2006). Detecting outliers when fitting data with nonlinear regression - a new method based on robust nonlinear regression and the false discovery rate. BMC Bioinform..

[50] Dai Y., Jia P., Fang Y., Liu H., Jiao X., He J.C., Ding X. (2016). miR-146a is essential for lipopolysaccharide (LPS)-induced cross-tolerance against kidney ischemia/reperfusion injury in mice. Sci. Rep..

[51] Sato F., Tsuchiya S., Terasawa K., Tsujimoto G. (2009). Intra-platform repeatability and inter-platform comparability of microRNA microarray technology. PLoS ONE.

[52] Git A., Dvinge H., Salmon-Divon M., Osborne M., Kutter C., Hadfield J., Bertone P., Caldas C. (2010). Systematic comparison of microarray profiling, real-time PCR, and next-generation sequencing technologies for measuring differential microRNA expression. RNA.

[53] Chen J., Cui Y., Zhang N., Yao X., Wang Z., Yang L. (2019). Oleanolic acid attenuated diabetic mesangial cell injury by activation of autophagy via miRNA-142-5p/PTEN signaling. Cytotechnology.

[54] Soltaninejad E., Nicknam M.H., Nafar M., Ahmadpoor P., Pourrezagholi F., Sharbafi M.H., Hosseinzadeh M., Foroughi F., Yekaninejad M.S., Bahrami T. (2015). Differential expression of microRNAs in renal transplant patients with acute T-cell mediated rejection. Transpl. Immunol..

[55] Ben-Dov I.Z., Muthukumar T., Morozov P., Mueller F.B., Tuschl T., Suthanthiran M. (2012). MicroRNA Sequence Profiles of Human Kidney Allografts With or Without Tubulointerstitial Fibrosis. Transplant. J..

[56] Kim K., Yang D.K., Kim S., Kang H. (2015). MIR-142-3p Is a Regulator of the TGFβ-Mediated Vascular Smooth Muscle Cell Phenotype. J. Cell. Biochem..

[57] Ichii O., Otsuka S., Sasaki N., Namiki Y., Hashimoto Y., Kon Y. (2012). Altered expression of microRNA miR-146a correlates with the development of chronic renal inflammation. Kidney Int..

[58] Zhu G., Pei L., Lin F., Yin H., Li X., He W., Liu N., Gou X. (2019). Exosomes from human-bone-marrow-derived mesenchymal stem cells protect against renal ischemia/reperfusion injury via transferring miR-199a-3p. J. Cell. Physiol..

[59] Delić D., Wiech F., Urquhart R., Gabrielyan O., Rieber K., Rolser M., Tsuprykov O., Hasan A.A., Krämer B.K., Baum P. (2020). Linagliptin and telmisartan induced effects on renal and urinary exosomal miRNA expression in rats with 5/6 nephrectomy. Sci. Rep..

[60] Wang C., Zhu G., He W., Yin H., Lin F., Gou X., Li X. (2019). BMSCs protect against renal ischemia-reperfusion injury by secreting exosomes loaded with miR-199a-5p that target BIP to inhibit endoplasmic reticulum stress at the very early reperfusion stages. FASEB J..

[61] Lino Cardenas C.L., Henaoui I.S., Courcot E., Roderburg C., Cauffiez C., Aubert S., Copin M.-C., Wallaert B., Glowacki F., Dewaeles E. (2013). miR-199a-5p Is upregulated during fibrogenic response to tissue injury and mediates TGFbeta-induced lung fibroblast activation by targeting caveolin-1. PLoS Genet..

[62] Howe M.D., Furr J.W., Munshi Y., Roy-O’Reilly M.A., Maniskas M.E., Koellhoffer E.C., D’Aigle J., Sansing L.H., McCullough L.D., Urayama A. (2019). Transforming growth factor-β promotes basement membrane fibrosis, alters perivascular cerebrospinal fluid distribution, and worsens neurological recovery in the aged brain after stroke. GeroScience.

[63] Wu C., Lv C., Chen F., Ma X., Shao Y., Wang Q. (2015). The function of miR-199a-5p/Klotho regulating TLR4/NF-κB p65/NGAL pathways in rat mesangial cells cultured with high glucose and the mechanism. Mol. Cell. Endocrinol..

[64] Zhu X., Li W., Li H. (2018). miR-214 ameliorates acute kidney injury via targeting DKK3 and activating of Wnt/ β-catenin signaling pathway. Biol. Res..

[65] Liu M., Liu L., Bai M., Zhang L., Ma F., Yang X., Sun S. (2018). Hypoxia-induced activation of Twist/miR-214/E-cadherin axis promotes renal tubular epithelial cell mesenchymal transition and renal fibrosis. Biochem. Biophys. Res. Commun..

[66] Denby L., Ramdas V., Lu R., Conway B.R., Grant J.S., Dickinson B., Aurora A.B., McClure J.D., Kipgen D., Delles C. (2014). MicroRNA-214 antagonism protects against renal fibrosis. J. Am. Soc. Nephrol..

[67] Bai M., Chen H., Ding D., Song R., Lin J., Zhang Y., Guo Y., Chen S., Ding G., Zhang Y. (2019). MicroRNA-214 promotes chronic kidney disease by disrupting mitochondrial oxidative phosphorylation. Kidney Int..

[68] Yuan X., Wang X., Chen C., Zhou J., Han M. (2017). Bone mesenchymal stem cells ameliorate ischemia/reperfusion-induced damage in renal epithelial cells via microRNA-223. Stem Cell Res. Ther..

[69] Go V., Bowley B.G.E., Pessina M.A., Zhang Z.G., Chopp M., Finklestein S.P., Rosene D.L., Medalla M., Buller B., Moore T.L. (2020). Extracellular vesicles from mesenchymal stem cells reduce microglial-mediated neuroinflammation after cortical injury in aged Rhesus monkeys. GeroScience.

[70] Liu X., Xu Y., Deng Y., Li H. (2018). MicroRNA-223 Regulates Cardiac Fibrosis after Myocardial Infarction by Targeting RASA1. Cell. Physiol. Biochem..

[71] Meschiari C.A., Ero O.K., Pan H., Finkel T., Lindsey M.L. (2017). The impact of aging on cardiac extracellular matrix. GeroScience.

[72] Shih N.Y., Li J., Karpitskii V., Nguyen A., Dustin M.L., Kanagawa O., Miner J.H., Shaw A.S. (1999). Congenital nephrotic syndrome in mice lacking CD2-associated protein. Science.

[73] Kim J.M., Wu H., Green G., Winkler C.A., Kopp J.B., Miner J.H., Unanue E.R., Shaw A.S. (2003). CD2-associated protein haploinsufficiency is linked to glomerular disease susceptibility. Science.

[74] Xia J., Worzfeld T. (2016). Semaphorins and Plexins in Kidney Disease. Nephron.

[75] Chen Y., Lin L., Tao X., Song Y., Cui J., Wan J. (2019). The role of podocyte damage in the etiology of ischemia-reperfusion acute kidney injury and post-injury fibrosis. BMC Nephrol..

[76] Guan F., Villegas G., Teichman J., Mundel P., Tufro A. (2006). Autocrine class 3 semaphorin system regulates slit diaphragm proteins and podocyte survival. Kidney Int..

[77] Ranganathan P., Jayakumar C., Mohamed R., Weintraub N.L., Ramesh G. (2014). Semaphorin 3A inactivation suppresses ischemia-reperfusion-induced inflammation and acute kidney injury. Am. J. Physiol. Renal Physiol..

[78] Park M., Kwon C.H., Ha H.K., Han M., Song S.H. (2020). RNA-Seq identifies condition-specific biological signatures of ischemia-reperfusion injury in the human kidney. BMC Nephrol..

