# Supplementary material for: Delayed Contralateral Nephrectomy Halted Post-Ischemic Renal Fibrosis Progression and Inhibited the Ischemia-Induced Fibromir Upregulation in Mice
Source: Biomedicines. 2021 Jul 14;9(7):815. doi: 10.3390/biomedicines9070815 (PMC8301422; doi:10.3390/biomedicines9070815)
Supplement: Supplementary file 1 [file biomedicines-09-00815-s001.zip › biomedicines-1069772-supplementary.pdf]

Supplementary Materials: Supplementary materials can be found at www.mdpi.com/xxx/s1.

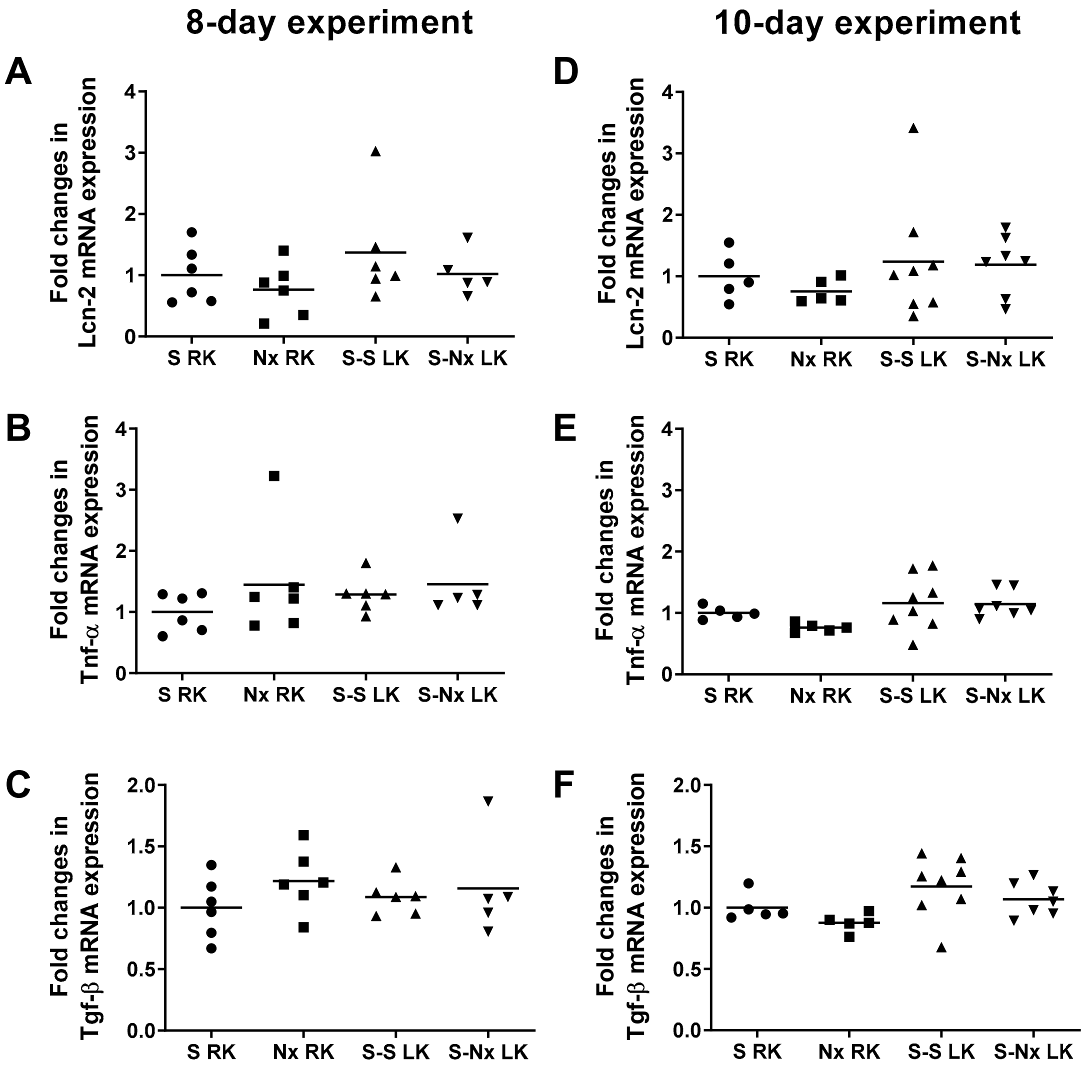

Supplementary Figure S1. Tnf-α, Tgf-β and Lcn-2 mRNA expression in the control kidney in the 8-day and 10-day experiments. S RK: right kidneys removed at the termination of the experiments, Nx RK: right kidneys removed at nephrectomy, S-S LK: sham-sham left kidneys, S-Nx LK: sham-Nx left kidneys. A-C: 8-day experiment, D-F: 10-day experiment. A, D: Lcn-2, B, E: Tnf-α, C, F: Tgf-β.

Supplementary Table S1. miRNAs upregulated at least 1.5-fold in the kidneys 8 days after IR based on the miRNA microarray (IR-S vs. S-S).

	miRNA	Fold change	p<		miRNA	Fold change	p<
1	mmu-miR-21a-5p	6.13±0.63	0.0000	23	mmu-miR-15b-5p	1.85±0.13	0.0000
2	mmu-miR-2137	5.31±1.88	0.0004	24	mmu-miR-34a-5p	1.83±0.29	0.0022
3	mmu-miR-142-3p	4.48±1.31	0.0001	25	mmu-miR-19a-3p	1.81±0.14	0.0000
4	mmu-miR-762	4.15±1.46	0.0005	26	mmu-miR-17-3p	1.76±0.22	0.0000
5	mmu-miR-223-3p	3.79±0.79	0.0000	27	mmu-miR-20a-5p	1.75±0.31	0.0006
6	mmu-miR-142-5p	3.58±0.81	0.0002	28	mmu-miR-1892	1.72±0.25	0.0000
7	mmu-miR-2861	3.08±0.94	0.0011	29	mmu-miR-489-5p	1.72±0.13	0.0000
8	mmu-miR-3102-5p	2.99±0.77	0.0001	30	mmu-miR-106a-5p	1.71±0.16	0.0000
9	mmu-miR-199a-5p	2.97±0.39	0.0000	31	mmu-miR-3095-3p	1.68±0.28	0.0002

10	mmu-miR-199a-3p/ mmu-miR-199b-3p	2.92±0.53	0.0000	32	mmu-miR-19b-3p	1.65±0.13	0.0001
11	mmu-miR-199b-5p	2.89±0.54	0.0002	33	mmu-miR-23a-3p	1.64±0.13	0.0000
12	mmu-miR-711	2.46±0.66	0.0146	34	mmu-miR-1894-3p	1.58±0.27	0.0010
13	mmu-miR-3473b	2.42±0.79	0.0300	35	mmu-miR-503-5p	1.54±0.23	0.0005
14	mmu-miR-214-3p	2.33±0.16	0.0000	36	mmu-miR-674-5p	1.53±0.17	0.0002
15	mmu-miR-146b-5p	2.27±0.64	0.0015	37	mmu-miR-25-5p	1.53±0.25	0.0006
16	mmu-miR-3970	2.20±0.31	0.0001	38	mmu-miR-27a-3p	1.53±0.16	0.0003
17	mmu-miR-146a-5p	2.15±0.45	0.0002	39	mmu-miR-665-3p	1.53±0.14	0.0000
18	mmu-miR-21a-3p	2.13±0.36	0.0000	40	mmu-miR-290a-5p	1.52±0.22	0.0009
19	mmu-miR-5129-5p	2.07±0.82	0.0283	41	mmu-miR-710	1.52±0.25	0.0029
20	mmu-miR-3100-3p	2.01±0.36	0.0000	42	mmu-miR-3084-3p	1.51±0.11	0.0004
21	mmu-miR-17-5p	1.87±0.12	0.0000	43	mmu-miR-24-2-5p	1.50±0.09	0.0002
22	mmu-miR-20b-5p	1.86±0.26	0.0001				

Supplementary Table S2. List of miRNAs downregulated at least 1.5-fold in the kidney 8 days after IR based on the miRNA microarray. (IR-S vs. S-S).

	miRNA	Fold change	p<		miRNA	Fold change	p<
1	mmu-miR-129-1-3p	0.31±0.06	0.0000	16	mmu-miR-30e-5p	0.62±0.06	0.0000
2	mmu-miR-193a-3p	0.42±0.08	0.0000	17	mmu-miR-378b	0.62±0.05	0.0000
3	mmu-miR-1839-3p	0.45±0.10	0.0000	18	mmu-miR-34b-3p	0.63±0.06	0.0000
4	mmu-miR-150-5p	0.47±0.05	0.0000	19	mmu-miR-505-5p	0.63±0.06	0.0000
5	mmu-miR-3961	0.48±0.06	0.0000	20	mmu-miR-669l-3p	0.63±0.11	0.0000
6	mmu-miR-190a-5p	0.48±0.04	0.0000	21	mmu-miR-192-5p	0.63±0.13	0.0005
7	mmu-miR-365-3p	0.51±0.09	0.0000	22	mmu-miR-30c-5p	0.64±0.03	0.0000
8	mmu-miR-194-5p	0.54±0.07	0.0002	23	mmu-miR-344h-3p	0.64±0.05	0.0000
9	mmu-miR-34c-3p	0.55±0.08	0.0000	24	mmu-miR-378a-3p/ mmu-miR-378b/ mmu-miR-378c	0.64±0.07	0.0000
10	mmu-miR-455-3p	0.56±0.04	0.0000	25	mmu-miR-1192	0.64±0.09	0.0002
11	mmu-miR-187-3p	0.57±0.05	0.0000	26	mmu-miR-378a-3p	0.65±0.07	0.0004
12	mmu-miR-29c-3p	0.57±0.09	0.0003	27	mmu-miR-215-5p	0.65±0.11	0.0014
13	mmu-miR-669m-3p	0.60±0.10	0.0000	28	mmu-miR-126a-5p	0.65±0.04	0.0001
14	mmu-miR-677-3p	0.62±0.05	0.0000	29	mmu-miR-30e-3p	0.66±0.06	0.0001
15	mmu-miR-185-5p	0.62±0.06	0.0003				

Supplementary Table S3. Validation of the miRNA miArray with qPCR. Statistical comparison of miRNA expression normalized to let-7g between the S-S, IR-S and IR-Nx groups.

	p, ANOVA significance	p, S-S vs. IR-S	p, IR-Nx vs. IR-S
miR-21a-5p	0.000	0.000	0.347
miR-2137	0.011	0.009	0.304
miR-142a-3p	0.000	0.000	0.946
miR-762	0.900	-	-
miR-223-3p	0.000	0.000	0.309
miR-142a-5p	0.000	0.000	0.940
miR-3102-5p	0.136	-	-
miR-199a-5p	0.000	0.001	0.420
miR-199a-3p	0.000	0.000	1.000
miR-214-3p	0.000	0.000	0.896
miR-146a-5p	0.000	0.001	0.990
miR-21a-3p	0.000	0.000	0.008
miR-129-1-3p	0.174	-	-

Supplementary Table S4. Comparison of miRNA expression normalized to let-7g between the S-S, S-Nx left kidneys and the control right kidneys.

	p, ANOVA significance	
	8-day experiment	10-day experiment
miR-21a-3p	0.078	0.668
miR-21a-5p	0.935	0.364
miR-142a-3p	0.379	0.756
miR-142a-5p	0.086	0.523
miR-146a-5p	0.328	0.974
miR-199a-3p	0.104	0.865
miR-199a-5p	0.321	0.559
miR-214-3p	0.166	0.837
miR-223-3p	0.297	0.588
